# Deregulation of N6-Methyladenosine RNA Modification and Its Erasers FTO/ALKBH5 among the Main Renal Cell Tumor Subtypes

**DOI:** 10.3390/jpm11100996

**Published:** 2021-09-30

**Authors:** Catarina Guimarães-Teixeira, Daniela Barros-Silva, João Lobo, Diana Soares-Fernandes, Vera Constâncio, Pedro Leite-Silva, Rui Silva-Santos, Isaac Braga, Rui Henrique, Vera Miranda-Gonçalves, Carmen Jerónimo

**Affiliations:** 1 Cancer Biology and Epigenetics Group, Research Center of IPO Porto (CI-IPOP)/RISE@CI-IPOP (Health Research Network), Portuguese Oncology Institute of Porto (IPO Porto)/Porto Comprehensive Cancer Center (Porto.CCC), Rua Dr. António Bernardino de Almeida, 4200-072 Porto, Portugal; catarina.guimaraes.teixeira@ipoporto.min-saude.pt (C.G.-T.); daniela.silva@ipoporto.min-saude.pt (D.B.-S.); jpedro.lobo@ipoporto.min-saude.pt (J.L.); diana.soares.fernandes@ipoporto.min-saude.pt (D.S.-F.); vera.salvado.constancio@ipoporto.min-saude.pt (V.C.); henrique@ipoporto.min-saude.pt (R.H.); vera.miranda.goncalves@ipoporto.min-saude.pt (V.M.-G.); 2 PhD Programme in Pathology & Molecular Genetics, School of Medicine & Biomedical Sciences, University of Porto (ICBAS-UP), Rua de Jorge Viterbo Ferreira, nº 228, 4050-313 Porto, Portugal; 3 Department of Pathology, Portuguese Oncology Institute of Porto (IPOP), Rua Dr. António Bernardino de Almeida, 4200-072 Porto, Portugal; rui.silva.santos@ipoporto.min-saude.pt; 4 Department of Pathology and Molecular Immunology, School of Medicine & Biomedical Sciences, University of Porto (ICBAS-UP), Rua de Jorge Viterbo Ferreira, nº 228, 4050-313 Porto, Portugal; 5 Cancer Epidemiology, Research Center of IPO Porto (CI-IPOP)/RISE@CI-IPOP (Health Research Network), Portuguese Oncology Institute of Porto (IPO Porto)/Porto Comprehensive Cancer Center (Porto.CCC), R. Dr. António Bernardino de Almeida, 4200-072 Porto, Portugal; pedro.silva@ipoporto.min-saude.pt; 6 Department of Urology, Portuguese Oncology Institute of Porto (IPOP), Rua Dr. António Bernardino de Almeida, 4200-072 Porto, Portugal; isaac.braga@gmail.com

**Keywords:** Epitranscriptomics, M^6^A, RNA, renal cell carcinoma, oncocytomas, FTO, ALKBH5

## Abstract

(1) Background: Methylation of N^6^-adenosine (m^6^A) is the most abundant messenger RNA (mRNA) modification in eukaryotes. We assessed the expression profiles of m^6^A regulatory proteins in renal cell carcinoma (RCC) and their clinical relevance, namely, as potential biomarkers. (2) Methods: In silico analysis of *The Cancer Genome Atlas* (TCGA) dataset was use for evaluating the expression of the m^6^A regulatory proteins among RCC subtypes and select the most promising candidates for further validation. ALKBH5 and FTO transcript and protein expression were evaluated in a series of primary RCC (*n* = 120) and 40 oncocytomas selected at IPO Porto. (3) Results: In silico analysis of TCGA dataset disclosed altered expression of the major m^6^A demethylases among RCC subtypes, particularly FTO and ALKBH5. Furthermore, decreased FTO mRNA levels associated with poor prognosis in ccRCC and pRCC. In IPO Porto’s cohort, FTO and ALKBH5 transcript levels discriminated ccRCC from oncocytomas. Furthermore, FTO and ALKBH5 immunoexpression differed among RCC subtypes, with higher expression levels found in ccRCC comparatively to the other RCC subtypes and oncocytomas. (4) Conclusion: We conclude that altered expression of m^6^A RNA demethylases is common in RCC and seems to be subtype specific. Specifically, FTO and ALKBH5 might constitute new candidate biomarkers for RCC patient management, aiding in differential diagnosis of renal masses and prognostication.

## 1. Introduction

Renal cell carcinoma (RCC) is the one of the most prevalent urological cancers in both genders, with 431,288 new cases and 179,368 deaths according to GLOBOCAN 2020 [1]. RCC is stratified into different subtypes, including clear cell renal cell carcinoma (ccRCC) (75–85%), papillary RCC (pRCC) (10–15%), chromophobe RCC (chRCC) (5–10%) and other less common entities, including collecting duct RCC and medullary RCC [2,3]. In contrast, renal oncocytomas are rather common benign tumors, which simulate RCC, thus constituting an important differential diagnosis [4].

Approximately 20–30% of RCC cases are diagnosed as disseminated disease, and about 20–40% of organ-confined RCCs will eventually progress to metastatic disease. Survival rate at 5 years in stage IV disease is only 12%, making RCC one of the deadliest urogenital neoplasms. Improvements in molecular understanding of RCC and identification of new biomarkers predictive of survival will refine treatment strategies and will be critical to the improvement of subtype-specific targeted therapies [5,6,7].

Epitranscriptomics focuses on RNA modifications, which have been implicated in many biological and pathological processes. Methylation of N^6^-adenosine (m^6^A) is the most abundant messenger RNA (mRNA) modification in eukaryotes influencing gene expression [8,9]. The m^6^A sites are enriched within the conserved motif containing DRACH sequence (D = A/G/U, R = A/G; H = A/C/U) and is found in the 5′-UTR, near the stop codon in the 3′-UTR and in long exons.

M^6^A is regulated by the RNA methyltransferase complex, RNA demethylases, and m^6^A readers. The heterodimeric core complex of RNA methylation comprises methyltransferase-like protein 3 (METTL3) and methyltransferase-like protein 14 (METTL14) and, additionally, the auxiliary proteins Wilms’ tumor 1-associated protein (WTAP), Virilizer-like m^6^A methyltransferase associated protein (VIRMA/KIAA1429) and RNA-binding protein 15 (RBM15). The family of proteins that acts as m^6^A demethylases includes fat mass and obesity-associated protein (FTO) and α-ketoglutarate-dependent dioxygenase alkB homologue 5 (ALKBH5) [10,11], which remove m^6^A modification from RNA.

M^6^A RNA modification is reversible and dynamic, being associated with multiple diseases including cancer. Specifically, it influences tumor cell proliferation, differentiation, tumorigenesis, invasion, and metastasis [12,13,14]. Moreover, this modification was shown to play a critical role in the progression of several cancers [15,16,17], including prostate [18], testis [19] and breast [20], as well as in acute myeloid leukemia [21].

Recently, m^6^A alterations were reported in RCC, more specifically, in ccRCC. Indeed, Wang and coworkers showed that decreased METTL14 expression induced tumor growth and associated with poor prognosis in ccRCC [22]. Furthermore, Li et al. evaluated METTL3 in RCC and demonstrated an oncogenic role, promoting tumor cell proliferation, migration and invasion [23]. Nonetheless, most of those studies focused on components of the methyltransferase complex. Contrarily, the role of erasers has been seldom explored in RCC. Furthermore, the few works available on m^6^A modification are “ccRCC-centric”, focusing mainly on understanding the implications of this modification in the most common RCC subtype [24,25,26,27,28,29]. Interestingly, the recent integrated molecular analyses of *The Cancer Genome Atlas* (TCGA) emphasized the metabolic deregulation in the several renal tumors, with erasers FTO and ALKBH5 specifically associated with metabolic reprogramming [30,31,32]. Furthermore, both erasers were also implicated in mitochondrial content regulation in RCC [33,34].

Herein, we aimed to access the expression of key m^6^A modulator enzymes-four writers (METTL3, METTL14, VIRMA, and WTAP) and two erasers (FTO and ALKBH5)-in the most common subtypes of renal cell tumors. Moreover, we conducted a comprehensive analysis using publicly available data on RCC subtypes from the TCGA dataset. We focused on erasers and assessed the respective mRNA and protein expression levels among the various RCC subtypes and oncocytoma, assessing their usefulness for tumor subtyping and prognostication.

## 2. Materials and Methods

We assess the differential expression of m^6^A erasers FTO and ALKBH5, in a cohort of RCC patients and oncocytomas, looking for clinical relevance of these findings.

### 2.1. In Silico Analysis

To evaluate the expression of the m^6^A regulatory proteins (writers and erasers) among RCC subtypes and select the most promising candidates for further validation, the online platform cBio-Portal was used [33] with the user-defined entry gene set “METTL3, METTL14, WTAP, VIRMA, FTO and ALKBH5”. *The Cancer Genome Atlas* (http://cancergenome.nih.gov (accessed on 30 April 2021)) databases for the three subtypes were selected and retrieved for different analyses. 

Overall (OS) and progression-free (PFS) survival considering standard clinical variables and mRNA expression of FTO and ALKBH5 were analyzed through computation of Kaplan-Meier curves and compared using log rank test. A Cox regression model (multivariable model) was performed to calculate hazard ratios (HR) and 95% CI, comprising significant clinicopathological variables (pathological stage and age at diagnosis) and categorized FTO and ALKBH5 expression status. Survival times were calculated from the date of the diagnosis to the date of last follow-up or death (OS) or to the date of last follow-up or progression (PFS). For this, all cases were coded based on FTO and ALKBH5 expression levels using the 25th and 75th percentile as empirical cutoff values, respectively.

### 2.2. Patients and Samples 

All patients presenting with renal cell tumors at the Portuguese Oncology Institute of Porto (IPO Porto) between 2001 and 2014 were retrospectively queried using the Department of Pathology’s database. Thus, a cohort of 120 RCC patients and 40 oncocytomas was chosen for this study (the first 40 cases of each subtype were selected). Tumor samples included fresh frozen tissue (for RNA extraction) and formalin-fixed paraffin embedded tissues (for immunohistochemistry) selected by an uropathologist. All samples were derived from primary renal cell tumors without any prior treatment. All patients were treated at IPO Porto by the same multidisciplinary team.

Clinical files and pathology reports were reviewed. All histological slides were reviewed by a dedicated uropathologist and tumors were reclassified considering the most recent 2016 World Health Organization (WHO) Classification of Tumors of the Urinary System and Male Genital Organs [35]. Staging was performed according to the 8th edition of the American Joint Committee on Cancer (AJCC) staging manual [36] (Supplementary Table S1).

This study was approved by the ethics committee (Comissão de Ética para a Saúde) of the Portuguese Oncology Institute of Porto (CES-IPO 321/020).

### 2.3. RNA Extraction, cDNA Synthesis and RT-qPCR

After serial sectioning fresh-frozen tumor samples were suspended in TRIzol^®^ reagent (Invitrogen^TM^, Cat. #15596018) and chloroform (Merk Millipore, Cat. #MCX10601) was added to the lysed cells. Total RNA was purified using the Ambion^®^ PureLink RNA Mini Kit (Invitrogen^TM^, Cat. #12183025), according to manufacturer’s recommendations. NanoDrop ND-1000 spectrophotometer (NanoDrop Technologies) was using for determined RNA concentrations and purity ratios.

cDNA synthesis and whole transcriptome amplification (WTA) was performed on a series of 160 samples. A total of 300 ng was reversely transcribed and amplified using TransPlex^®^ Whole Transcriptome Amplification Kit (Sigma-Aldrich^®^ Cat. #WTA1) purified with QIAquick PCR Purification Kit (QIA-GEN, Cat. #28106). The reaction was performed using the following conditions: 5 min at 95 °C, 30 cycles × (20 sec at 94 °C and 5 min at 65 °C). Samples were then stored at − 20 °C. 

Real-time quantitative Polymerase Chain Reaction (RT-qPCR) was performed in 384-well plates in QuantStudio 12K Flex Real-Time PCR System (Thermo Fisher, Foster, CA, USA) according to manufacturer’s recommendations. Reactions were run at 60 °C for 45 cycles. Serial dilutions of cDNA obtained from an RNA pool of multiple cell lines were used to compute standard curves for each plate, which were used to guarantee interpolate reaction efficiency. All experiments were run in triplicate, and two no-template controls were included in each plate. 

FTO and ALKBH5 mRNA levels were evaluated using TaqMan^®^ Gene Expression Assays (Applied Biosystems^®^ Cat. #4351370). As a housekeeping gene for normalization, the GUSB TaqMan^®^ Gene Expression Assay (Bio-Rad: qHsaCIP0028142) was used. Results were reported as: Relative levels (target) = Mean quantity (target)/Mean quantity (GUSB) × 1000, for easier tabulation.

### 2.4. Immunohistochemical Analysis

Immunohistochemistry (IHC) for m^6^A modification and erasers ALKBH5 and FTO was performed using the Novolink^TM^ Max Polymer Detection System (Leica Biosystems, Germany). Four micrometer thick sections were cut from formalin-fixed paraffin embedded samples (matching the frozen sections) and placed on coated slides. The procedure was performed as previously described in [19]. Incubation with the primary antibody was performed at RT for 1h (FTO ab92821, dilution 1:500; ALKBH5 16837-1-AP, dilution 1:1000; m^6^A ab190886, dilution 1:750). Normal testis parenchyma and normal brain tissue were used as positive controls for FTO/ALKBH5 and m^6^A, respectively.

Semi-quantitative immunoexpression analysis was performed by an experienced uropathologist and categorized according to intensity and percentage of stained cells in the slide (between 0–100%). The following scores were used for further analysis: nuclear intensity score, consisting of score 0 (absent immunoexpression), score 1+ (immunoexpression only barely discernible at high power magnification), score 2+ (immunoexpression well discernible at high power but faint in low power magnification), and score 3+ (strong immunoexpression well discernible at low power magnification); and nuclear percentage score, consisting of score 0 (<1% of immunoreactive cells), score 1+ (<40% of immunoreactive cells), score 2+ (40–80% of immunoreactive cells) and score 3+ (80–100% of immunoreactive cells). The final staining score was calculated by multiplying intensity and percentage scores, resulting in a combined score value ranging from 0 to 9+. 

### 2.5. Statistical Analysis

Statistical analysis was performed using the GraphPad Prim 9.0 software (GraphPad Software Inc., Chicago IL, USA) and IBM^®^ SPSS^®^ Statistic software version 23 (IBM-SPSS Inc., La Jolla, CA; USA). Non-parametric Mann-Whitney U-tests or Kruskal-Wallis test were used to compare the distribution of continuous variables among groups. Bonferroni’s or Dunn’s corrections were employed in case of multiple testing, as appropriate. Associations between categorical variables were assessed using Chi square and Fisher’s exact test. Correlation between continuous variables was assessed with the non-parametric Spearman’s correlation test. ROC curve analyses were performed for assessing the discrimination performance of FTO and ALKBH5 transcript levels among renal tumor subtypes as described in [19].

The *p*-values were considered statistically significant when less than 0.05. Significance is shown and depicted as follows: * *p* ≤ 0.05, ** *p* < 0.01, *** *p* < 0.001, **** *p* < 0.0001 and ^ns^ *p* > 0.05 (non-significant).

## 3. Results

### 3.1. In Silico Analysis of m^6^A-Related Proteins in TCGA’s RCC Patients

In silico analysis of the publicly available The Cancer Genome Atlas (TCGA) database, accessible for analysis at cBioPortal, concerning m^6^A-related proteins (writers and erasers) was carried out [37]. The TCGA dataset included tumor samples from 352 patients with ccRCC, 271 patients with pRCC and 65 patients with chRCC. 

**Figure 1:**
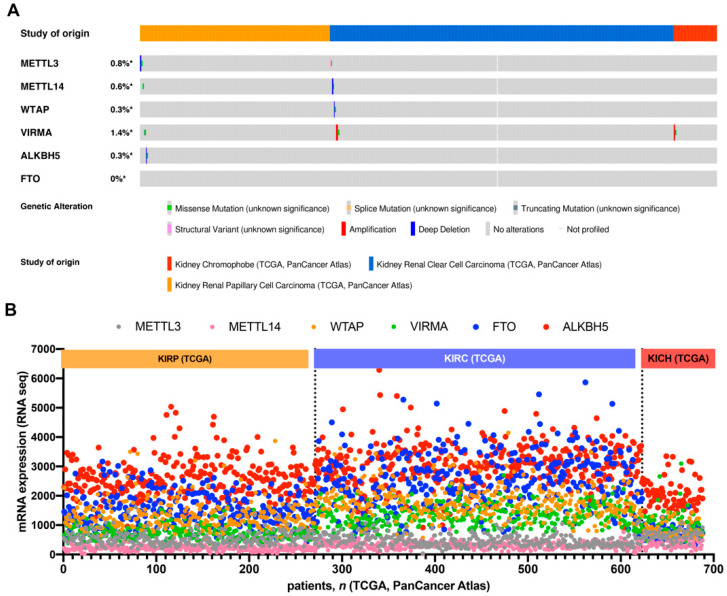
In silico analysis of mRNA expression: (**A**) alterations frequency of m^6^A regulatory proteins in RCC TCGA cohort. (**B**) Differential mRNA expression of several players. Notice the high expression of FTO and ALKBH5 compared to other queried genes. Abbreviations: METTL3—Methyltransferase-like protein 3, METTL14—methyltransferase-like protein 14, WTAP—Wilms’ tumor 1-associated protein, VIRMA—Virilizer-like, FTO—fat mass and obesity-associated protein, ALKBH5—α-ketoglutarate-dependent dioxygenase alkB homologue 5. * Ratio between patients presenting alterations and total number of patients included in the study.

Overall, analysis of the genomic regions encoding m^6^A players disclosed no (FTO) or less than 1% (METTL3, METLL14, WTAP and ALKBH5) genomic alterations in RCC, except for VIRMA, which disclosed an amplification frequency of 1.4% (Figure 1A). 

We then explored mRNA expression of the several players among different tumor subtypes and found that erasers, FTO and ALKBH5, were expressed at higher levels, compared to writers. Additionally, most players (WTAP, VIRMA, FTO and ALKBH5) were expressed at lower levels in chRCC compared to ccRCC and pRCC, with ccRCC displaying the highest FTO and ALKBH5 transcript levels among RCC subtypes (Figure 1B). 

Interestingly, in univariable analysis, higher FTO expression associated with better overall and progression free survival both in ccRCC and pRCC patients, whereas no associations were disclosed concerning ALKBH5 expressions levels (Supplementary Tables S2–S4 and Supplementary Figure S1). Importantly, in multivariable analysis, higher (>P25) FTO expression had a protective effect for pRCC patients progression as well as for overall survival in ccRCC and pRCC patients, whereas more advanced pathological stages associated with worse prognoses, as well as age at diagnosis, but only for overall survival in ccRCC patients (Table 1). 

Considering the higher relative expression levels of erasers compared to writers in this in silico analysis, erasers FTO and ALKBH5 were further investigated in a separate (*IPO Porto*’s) RCC patient cohort, to evaluate the potential clinical significance of those findings.

### 3.2. Differential FTO and ALKBH5 Expression among RCC Subtypes and Oncocytoma in IPOPorto’s Cohort

A total of 120 RCC (ccRCC, pRCC and chRCC, 40 cases per subtype) as well as 40 renal oncocytomas from patients surgically treated at IPO Porto, were included in this study. Patients’ age varied from 28 to 86 years old (Supplementary Table S1). For purposes of validation of our cohort, we demonstrated that patients with higher disease pathological stage at presentation experienced poorer overall-survival, disease-free survival, and disease-specific survival (*p* = 0.002, *p* = 0.0008 and *p* = 0.0015, respectively) compared to lower stage disease (Supplementary Figure S2). These results confirm the representativity of this independent RCC cohort.

**Figure 2:**
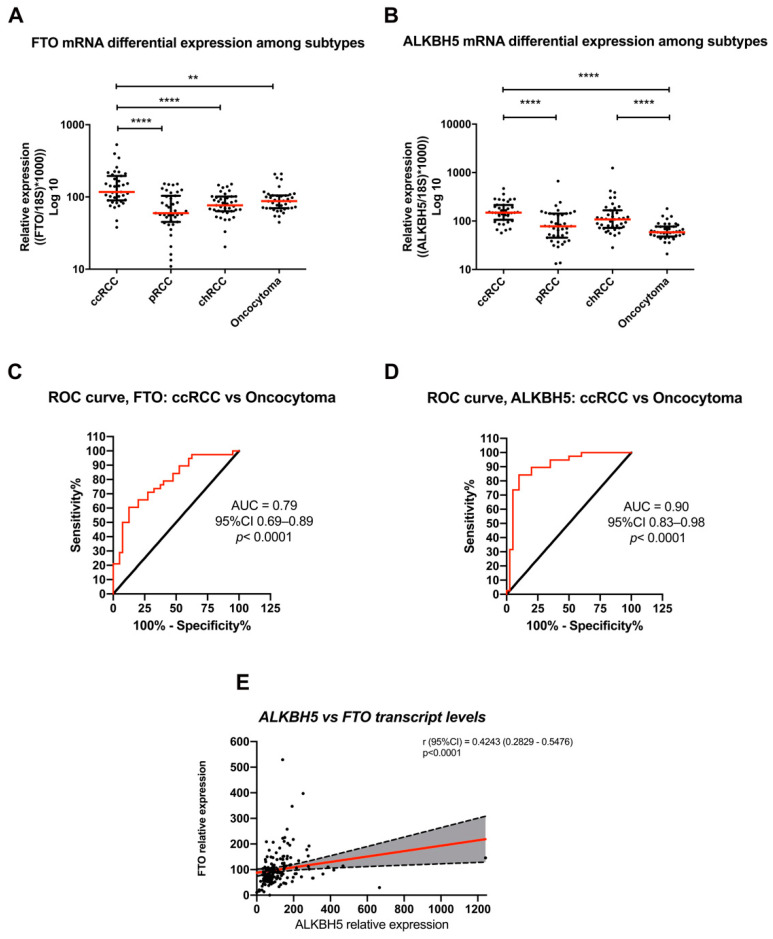
Transcript levels of FTO and ALKBH5 in IPO Porto’s Cohort: (**A**) FTO mRNA expression in all RCC subtypes and oncocytomas (**B**) ALKBH5 mRNA expression in all RCC subtypes and oncocytomas (**C**,**D**) ROC curve for discrimination among ccRCC and oncocytoma based on FTO and ALKBH5 mRNA expression levels, respectively. ROC, receiver operating characteristic; AUC, area under the curve. (**E**) Correlation between mRNA expression levels of FTO and ALKBH5. Data is normalized for reference gene GUSB. Abbreviations: AUC area under the curve; CI confidence interval; ccRCC—clear cell renal cell carcinoma; pRCC—papillary renal cell carcinoma; chRCC—chromophobe renal cell carcinoma; Statistically significant p-value: ** *p* < 0.01, **** *p* < 0.0001.

FTO and ALKBH5 transcript levels were significantly higher in ccRCC compared to oncocytoma (*p* = 0.0088 and *p* < 0.0001, respectively). Among RCC subtypes, FTO and ALBH5 mRNA expression levels were significantly higher in ccRCC compared to pRCC and chRCC (*p* < 0.0001), whereas pRCC depicted the lowest expression levels (*p* < 0.0001) (Figure 2A,B). 

We then assessed whether FTO and ALKBH5 transcript levels discriminated among renal cell tumor subtypes, using ROC curve analysis. Interestingly, ccRCC was discriminated from oncocytomas with an AUC of 0.79 and 0.90 (Figure 2C,D), but no additional statistically significant differences were observed for the remaining comparisons (Supplementary Figures S3 and S4).

Remarkably, when examining all tumor samples, FTO and ALKBH5 mRNA expression levels were positively correlated (r_s_ = 0.4243, *p* < 0.001) (Figure 2E).

### 3.3. Evaluation of FTO, ALKBH5 and m^6^A Immunoexpression in Primary Tumors

**Figure 3:**
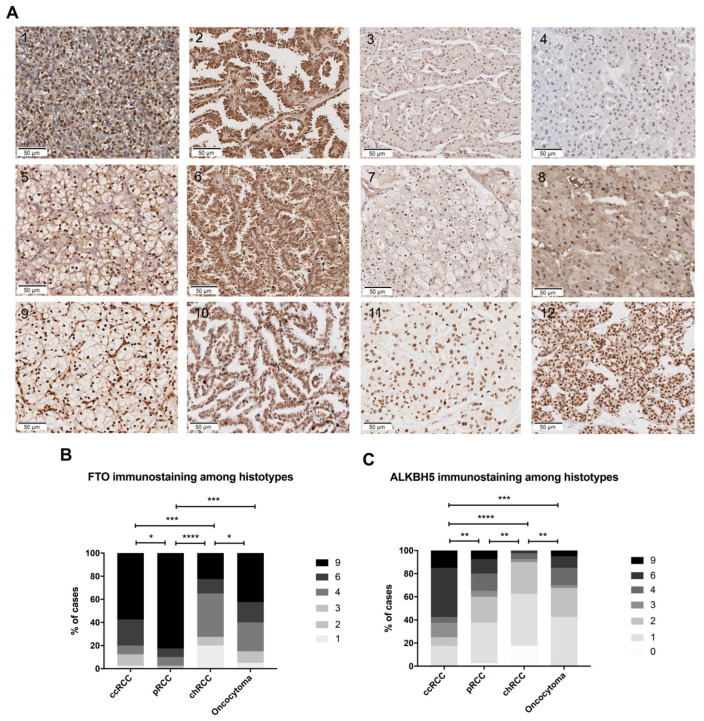
Differential immunoexpression of FTO and ALKBH5: (**A**) illustrative examples of FTO (upper row), ALKBH5 (middle row) and m^6^A (lower row) immunoexpression (left to right: ccRCC, pRCC, chRCC and oncocytomas) (**B,C**) Comparison between the immunoexpression of FTO and ALKBH5 in all RCC subtypes and oncocytomas. Detailed p-values for each comparison: FTO adjusted p-value of 0.0164 (pRCC/ccRCC), 0.0002 (ccRCC/chRCC), <0.0001 (pRCC/chRCC), 0.0001 (pRCC/oncocytomas) and 0.0203 (chRCC/oncocytomas); ALKBH5 adjusted p-value of 0.0017 (pRCC/ccRCC), <0.0001 (ccRCC/chRCC), 0.0002 (ccRCC/oncocytoma), 0.0011 (pRCC/chRCC) and 0.0041 (chRCC/oncocytomas). Immunostaining based on h-score (ranges from 0, +1, +2, +3, +4, +6, +9). Abbreviations: ccRCC—clear cell renal cell carcinoma; pRCC—papillary renal cell carcinoma; chRCC—chromophobe renal cell carcinoma; Statistically significant *p*-value: * *p* < 0.05, ** *p* < 0.01, *** *p* < 0.001, **** *p* < 0.0001.

M^6^A modification, FTO and ALKBH5 differed in cellular distribution between tumor samples. The m^6^A immunostaining was predominantly nuclear with cytoplasmic staining in only 3% of the cases. Regarding m^6^A regulators, FTO staining was predominantly nuclear, whereas ALKBH5 exhibited both nuclear and cytoplasmic staining in most cases (illustrative examples of immunostaining are shown in Figure 3A). 

FTO and ALKBH5 immunostaining significantly differed between RCC subtypes and oncocytomas (Figure 3B, including detailed statistical analysis). Overall, both erasers disclosed lower expression in oncocytomas compared to RCC subtypes. Among RCCs, and in contrast with transcript information, chRCC disclosed the lowest FTO and ALKBH5 expression, whereas immunoexpression scores were remarkably higher in pRCC and ccRCC. Nonetheless, no significant differences were apparent regarding m^6^A immunostaining among RCC subtypes (Figure 3C).

### 3.4. Association with Clinicopathological Parameters and Chromosomal Aberrations

In RCCs, no statistically significant associations were disclosed between FTO and ALKBH5 imunoexpression, neither with nuclear grade nor pathological stage. In RCTs, imunoexpression of both proteins did not associate with gender, but significantly associated with patients’ age (p<0.0001, for both proteins). Moreover, and contrary to disease stage (Figure 3), no significant associations were found between FTO and ALKBH5 imunoexpression and patient survival (Supplementary Figure S5). 

Because copy number variation (CNV) might have impacted in the observed expression changes, we re-analyzed the FISH data previously published for our cohort [38]. FTO and ALKBH5 are located at chromosomes 16 and 17, respectively. We found the absence of chromosomal deletions or duplications in patients with low or high expression levels, respectively. Thus, FTO and ALKBH5 expression alterations found among different RCC subtypes and oncocytomas do not seem to derive from copy number variations.

## 4. Discussion

RCC is one of the most common urological cancers worldwide and although most patients experience a favorable survival outcome, the 5-year survival rate for patients with metastatic disease does not exceed 12%. The mechanisms associated with advanced disease are still poorly understood, and novel, more effective, targeted treatments are needed. Furthermore, the initial diagnosis of patients with renal masses is challenging because a definitive characterization is only possible upon histological assessment of the nephrectomy specimen. Thus, new biomarkers may improve RCC diagnosis and subtyping, as well as prediction of disease progression. Moreover, investigation of new biomarkers may perfect patient monitoring and identify novel targets for more effective therapies [6,39,40,41]. 

RNA modifications, an additional regulatory layer of biology, constitute the “Epitranscriptome”. There are currently more than 170 RNA base modifications, the majority of these already reported on mRNA, lncRNA, tRNA and rRNA. RNA chemical alterations can directly affect cell biology regulation, RNA stability, localization, splicing and translation of both coding and non-coding transcripts [42,43,44]. M^6^A is the most prevalent modification in mammals and its deposition is accomplished by a m^6^A methylome complex [45,46]. An important discovery was the finding of enzymes that can demethylate m^6^A. The first identified m^6^A demethylase was FTO, which is conserved among eukaryotes. ALKBH5 is another recently acknowledged demethylase, which affects the export of nuclear RNA [47]. Moreover, ALK homologues 1–8 and FTO have been shown to repair several different DNA and RNA lesions. Thus, beyond the demethylating function, the ALK family and FTO were shown to have a broader biological function, being also implicated in tumor chemoresistance [48,49]. 

FTO overexpression in bladder cancer correlated with poor prognosis indicating a potential oncogenic function [50]. This player was also found as a regulator of metabolic diseases, as well as in human obesity [51,52]. Similarly, an oncogenic role has been suggested for ALKBH5, as its knockdown inhibited lung tumorigenesis [53] and gastric cancer invasion and metastasis [54]. Moreover, ALKBH5 was also demonstrated to regulate cardiomyocyte proliferation [55]. Importantly, as already described, these players may constitute potential therapeutic targets, which makes their expression levels potential predictive biomarkers [56,57]. 

Considering the role of m^6^A modification and its erasers in carcinogenesis, we hypothesized that demethylating enzymes, FTO and ALKBH5, might be potential biomarkers with distinct roles in different RCC subtypes and oncocytomas. In particular, the link of several RCC subtypes to specific metabolic pathways, and the known influence of erasers in regulating metabolic players, suggests a relevant and differential role of these erasers in renal cell tumors.

Firstly, we surveyed the TCGA dataset to screen for the best candidates among all m^6^A regulatory proteins. Remarkably, in RCC subtypes, all m^6^A regulators disclosed very few copy number alterations or mutations. Thorough evaluation of the global expression of all regulators demonstrated that erasers (FTO and ALKBH5) are highly expressed in RCC compared to writers (METTL3/14, WTAP and VIRMA), specifically in ccRCC, prompting further investigation in an independent patient (IPO Porto) cohort. Furthermore, high FTO transcript levels revealed a protective effect for PFS in pRCC and OS in ccRCC and pRCC, independently of other relevant clinical and pathological variables in TCGA dataset. Interestingly, ccRCC displayed higher FTO and ALKBH5 mRNA levels compared to other RCC subtypes, which were able to discriminate ccRCC from oncocytomas.

Importantly, the observed altered expression of both erasers in our cohort was not due to copy number variations [38], suggesting alternative regulatory mechanisms for gene expression. Overall, these results were also partially concordant with immunoexpression analysis, as ccRCC also showed significantly higher FTO and ALKBH5 immunoscores. Nonetheless, in pRCC, mRNA and protein expression data for both erasers were not concordant (high protein expression but low transcript levels). We hypothesize that this might be due to post-transcriptional regulatory mechanisms in pRCC [56,57,58], requiring confirmation. It is noteworthy that pRCC are remarkably heterogeneous, with both type I and II tumors comprising distinct molecular backgrounds, which may further hinder expression analyses. Importantly, a positive correlation between the transcript levels of the two erasers was demonstrated, indicating that they possibly cooperate in accomplishing m^6^A demethylation. 

Interestingly, similar m^6^A levels were found among the different RCC subtypes and oncocytomas. These observations may be explained by the fact that immunohistochemistry only allows for semi-quantitative assessment of m^6^A at the global level, whereas m^6^A modification may affect different transcripts (with distinct implications) among the various renal cell tumor subtypes. Furthermore, immunoexpression of both erasers was not observed exclusively in the nucleus, suggesting that demethylation may also occur in the cytoplasm or that the player may be assuming an alternative function, as previously suggested [25]. Indeed, these players may be involved in other biological/cellular processes, as already described [50,55].

Curiously, our results on m^6^A eraser expression are in line with those reported for other cancers, in which FTO and ALKBH5 were also found to be overexpressed [54,58,59,60,61]. Nonetheless, in the same line as *Strick* and collaborators, no associations were found between ALKBH5 or FTO expression and standard clinicopathological parameters, including nuclear grade and pathological stage, and neither ALKBH5 nor FTO protein expression were independent predictors of patient survival in our cohort. However, the same authors reported a reduced expression of these players in ccRCC compared to benign and normal tissue samples [25]. It should be noted, however, that they used tissue micro arrays which represent only very small portions of the tumor tissue, whereas we assessed immunoexpression in whole tissue slides. Moreover, they used “normal” parenchyma adjacent to tumors for comparisons, that due to the acknowledged phenomenon of “field effect”, may harbor molecular and epigenetic, as well as epitranscriptomic, alterations [62,63]. Herein, we did not include normal tissues in the analysis as we focused on the discrimination among major RCT subtypes. Finally, the analyzed tissue set was smaller than ours, with scanter representation of the several tumor types. Indeed, in our study there is a larger representation of the main RCC subtypes as well as oncocytomas (*n* = 40 for each). Furthermore, although demethylases’ expression heterogeneity was observed within each subtype, significant differences in FTO and ALKBH5 expression were found among the main tumor subtypes [25]. 

Remarkably, our results are in accordance with those of Xiao et al., who reported that high FTO expression in ccRCC correlated with increased tumor severity and poor patient survival [64]. Contrarily, Zhuang et al., through FTO knockdown in ccRCC cells, observed increased proliferation and decreased apoptosis [32]. Nonetheless, the role of FTO in the pathobiology of renal neoplasia remains elusive. Therefore, dedicated investigations, using in vitro and in vivo pre-clinical models, and larger well-defined patient cohorts are needed. The same is valid for ALKBH5, with only one study on RCC suggesting an oncogenic role for ALKBH5, as patients with high expression endured poor overall survival [31]. Interestingly, in our cohort, we disclosed high transcript and protein expression in ccRCC, however, there are no significant differences in further survival analysis.

Although the limited number of RCC-related deaths and progression events in our cohort impaired survival analysis, this cohort is similar, in many aspects, to TCGA dataset and it also reflects RCC epidemiology. Importantly, all tissues were evaluated in a single institution by the same multidisciplinary team entailing homogeneity in pathological scores assessment as well as therapeutic decisions.

## 5. Conclusions

In summary, we showed that FTO and ALKBH5 are differentially expressed among different RCC subtypes and oncocytomas, eventually proving useful for discrimination between malignant and benign renal cell tumors, as well as for prognostic assessment. Furthermore, a positive correlation between the two erasers was also observed, suggesting cooperation at molecular level.

These results emphasize the important role of RNA demethylases in RCC, confirming the in silico analysis. To the best of our knowledge, this is the first study focusing on the impact of m^6^A erasers in the major renal cell tumor subtypes. 

## Figures and Tables

**Table 1:** Cox regression analysis assessing the potential of clinical and FTO and ALKBH5 expression in the prediction of Progression-Free survival and Overall Survival. Abbreviations: HR—Hazard ratio; CI—Confidence interval; ccRCC—clear cell renal cell carcinoma; pRCC—papillary renal cell carcinoma; Statistically significant *p*-values (*p* < 0.05).

Progression-Free Survival (PFS)		HR	95% CI for HR	*p*-Value	HR	95% CI for HR	*p*-Value
Variable		ccRCC			pRCC		
FTO expression	≤P25	1.00	-	-	1.00	-	-
	>P25	0.67	0.43–1.05	0.081	0.42	0.24–0.75	0.004
Pathological stage	pT1	1.00	-	-	1.00	-	-
	pT2	3.08	1.59–5.95	<0.001	3.58	1.59–8.04	0.020
	pT3/4	5.96	3.54–10.05	<0.001	7.18	3.82–13.50	<0.001
Overall Survival (OS)		HR	95% CI for HR	*p*-Value	HR	95% CI for HR	*p*-Value
Variable		ccRCC			pRCC		
FTO expression	≤P25	1.00	-	0.035	1.00	-	-
	>P25	0.62	0.40–0.97	<0.001	0.48	0.24–0.97	0.040
Age at diagnosis	-	1.05	1.03–1.07	<0.001	-	-	-
Pathological stage	pT1	1.00	-	-	1.00	-	-
	pT2	1.25	0.62–2.53	0.5298	3.34	1.21–9.21	0.020
	pT3/4	3.24	2.04–5.13	<0.001	8.25	3.82–17.79	<0.001

## Data Availability

The datasets used and/or analyzed during the current study are available from the corresponding author on reasonable request.

## Supplementary Materials

The following are available online at https://www.mdpi.com/article/10.3390/jpm11100996/s1, Table S1: Clinicopathological characteristics of IPO Porto cohort; Table S2: Univariable analysis in TCGA ccRCC patients; Table S3: Univariable analysis in TCGA pRCC patients. Table S4: Univariable analysis in TCGA chRCC patients; Figure S1: Survival analysis in TCGA’s patients for ALKBH5 (A) Overall-survival (left) and Progression free-survival (right) in ccRCC (B) Overall-survival (left) and Progression free-survival (right) in pRCC (C) Overall-survival (left) and Progression free-survival (right) in chRCC and FTO (D) Overall-survival (left) and Progression free-survival (right) in ccRCC (E) Overall-survival (left) and Progression free-survival (right) in pRCC (F) Overall-survival (left) and Progression free-survival (right) in chRCC; Figure S2: Kaplan-Meier estimated (A) Disease-specific survival (B) Disease-free survival (C) Overall survival for stage of IPO Porto’s cohort; Figure S3: ROC curve for discrimination among different subtypes based on FTO mRNA expression levels in IPOPorto’s cohort. Abbreviations: ccRCC—clear cell renal cell carcinoma; pRCC—papillary renal cell carcinoma; chRCC—chromophobe renal cell carcinoma; AUC—Area under the curve; CI—Confidence interval; Figure S4: Transcript levels of ALKBH5: ROC curve for discrimination among different subtypes based on ALKBH5 mRNA expression levels in IPO Porto’s cohort. Abbreviations: ccRCC—clear cell renal cell carcinoma; pRCC—papillary renal cell carcinoma; chRCC—chromophobe renal cell carcinoma; AUC—Area under the curve; CI—Confidence interval. Figure S5: Kaplan-Meier estimated (A) Overall-survival in ccRCC (B) Overall-survival in chRCC (C) Overall survival in pRCC for FTO (right) and ALKBH5 (left) immunoexpression in IPO Porto cohort.

## Abbreviations

AJCC	American Joint Committee on Cancer
AUC	area under the curve
ccRCC	clear cell renal cell carcinoma
cDNA	complementary DNA
chRCC	chromophobe renal cell carcinoma
FFPE	formalin-fixed paraffin-embedded
GUSB	beta-glucoronidase
IHC	immunohistochemistry
m^6^A	N-6-methyladenosine
mRNA	messenger RNA
OR	odds ratio
pRCC	papillary renal cell carcinoma
PPV	positive predictive value
RNA	ribonucleic acid
ROC	receiver operating characteristics
rs	Spearman’s correlation coefficient
RT-qPCR	real-time quantitative polymerase chain reaction
TCGA	The Cancer Genome Atlas
WHO	World Health Organization

## References

[1] Sung H., Ferlay J., Siegel R.L., Laversanne M., Soerjomataram I., Jemal A., Bray F. (2021). Global cancer statistics 2020: GLOBOCAN estimates of incidence and mortality worldwide for 36 cancers in 185 countries. CA Cancer J. Clinicians.

[2] Haake S.M., Rathmell W.K. (2017). Renal cancer subtypes: Should we be lumping or splitting for therapeutic decision making?. Cancer.

[3] Hsieh J.J., Purdue M.P., Signoretti S., Swanton C., Albiges L., Schmidinger M., Heng D.Y., Larkin J., Ficarra V. (2017). Renal cell carcinoma. Nat. Rev. Dis. Primers.

[4] Williams G.M., Lynch D.T. (2021). Renal Oncocytoma.

[5] Linehan W.M., Ricketts C.J. (2019). The Cancer Genome Atlas of renal cell carcinoma: Findings and clinical implications. Nat. Rev. Urology.

[6] Padala S.A., Barsouk A., Thandra K.C., Saginala K., Mohammed A., Vakiti A., Rawla P., Barsouk A. (2020). Epidemiology of Renal Cell Carcinoma. World J. Oncol..

[7] Keizman D., Gottfriedm M., Ish-Shalom M., Maimon N., Peer A., Neumann A., Hammers H., Eisenberger M.A., Sinibaldi V., Pili R. (2014). Active smoking may negatively affect response rate, progression-free survival, and overall survival of patients with metastatic renal cell carcinoma treated with sunitinib. Oncologist.

[8] Lobo J., Barros-Silva D., Henrique R., Jeronimo C. (2018). The Emerging Role of Epitranscriptomics in Cancer: Focus on Urological Tumors. Genes.

[9] Zhao W., Qi X., Liu L., Ma S., Liu J., Wu J. (2020). Epigenetic Regulation of m(6)A Modifications in Human Cancer. Mol. Ther. Nucleic Acids.

[10] Lan Q., Liu P.Y., Haase J., Bell J.L., Huttelmaier S., Liu T. (2019). The Critical Role of RNA m(6)A Methylation in Cancer. Cancer Res..

[11] Yang S., Wei J., Cui Y.H., Park G., Shah P., Deng Y., Aplin A.E., Lu Z., Hwang S., He C. (2019). M(6)A mRNA demethylase FTO regulates melanoma tumorigenicity and response to anti-PD-1 blockade. Nat. Commun..

[12] Zhu W., Wang J.Z., Xu Z., Cao M., Hu Q., Pan C., Guo M., Wei J.F., Yang H. (2019). Detection of N6methyladenosine modification residues (Review). Int. J. Mol. Med..

[13] Wang T., Kong S., Tao M., Ju S. (2020). The potential role of RNA N6-methyladenosine in Cancer progression. Mol. Cancer.

[14] Jiang X., Liu B., Nie Z., Duan L., Xiong Q., Jin Z., Yang C., Chen Y. (2021). The role of m6A modification in the biological functions and diseases. Signal. Transduct. Target. Ther..

[15] Ma J.Z., Yang F., Zhou C.C., Liu F., Yuan J.H., Wang F., Wang T.T., Xu Q.G., Zhou W.P., Sun S.H. (2017). METTL14 suppresses the metastatic potential of hepatocellular carcinoma by modulating N(6) -methyladenosine-dependent primary MicroRNA processing. Hepatology.

[16] Cui Q., Shi H., Ye P., Li L., Qu Q., Sun G., Sun G., Lu Z., Huang Y., Yang C.G. (2017). M(6)A RNA Methylation Regulates the Self-Renewal and Tumorigenesis of Glioblastoma Stem Cells. Cell Rep..

[17] Weng H., Huang H., Wu H., Qin X., Zhao B.S., Dong L., Shi H., Skibbe J., Shen C., Hu C. (2018). METTL14 Inhibits Hematopoietic Stem/Progenitor Differentiation and Promotes Leukemogenesis via mRNA m(6)A Modification. Cell Stem cell.

[18] Barros-Silva D., Lobo J., Guimarães-Teixeira C., Carneiro I., Oliveira J., Martens-Uzunova E.S., Henrique R., Jerónimo C. (2020). VIRMA-Dependent N6-Methyladenosine Modifications Regulate the Expression of Long Non-Coding RNAs CCAT1 and CCAT2 in Prostate Cancer. Cancers.

[19] Lobo J., Costa A.L., Cantante M., Guimaraes R., Lopes P., Antunes L., Braga I., Oliveira J., Pelizzola M., Henrique R. (2019). M(6)A RNA modification and its writer/reader VIRMA/YTHDF3 in testicular germ cell tumors: A role in seminoma phenotype maintenance. J. Transl. Med..

[20] Wu L., Wu D., Ning J., Liu W., Zhang D. (2019). Changes of N6-methyladenosine modulators promote breast cancer progression. BMC Cancer.

[21] Ianniello Z., Fatica A. (2018). N6-Methyladenosine Role in Acute Myeloid Leukaemia. Int. J. Mol. Sci..

[22] Wang Y., Cong R., Liu S., Zhu B., Wang X., Xing Q. (2021). Decreased expression of METTL14 predicts poor prognosis and construction of a prognostic signature for clear cell renal cell carcinoma. Cancer Cell Int..

[23] Li X., Tang J., Huang W., Wang F., Li P., Qin C., Qin Z., Zou Q., Wei J., Hua L. (2017). The M6A methyltransferase METTL3: Acting as a tumor suppressor in renal cell carcinoma. Oncotarget.

[24] Chen J., Yu K., Zhong G., Shen W. (2020). Identification of a m(6)A RNA methylation regulators-based signature for predicting the prognosis of clear cell renal carcinoma. Cancer Cell Int..

[25] Strick A., Von Hagen F., Gundert L., Klümper N., Tolkach Y., Schmidt D., Kristiansen G., Toma M., Ritter M., Ellinger J. (2020). The N(6) -methyladenosine (m(6) A) erasers alkylation repair homologue 5 (ALKBH5) and fat mass and obesity-associated protein (FTO) are prognostic biomarkers in patients with clear cell renal carcinoma. BJU Int..

[26] Zhang Q.J., Luan J.C., Song L.B., Cong R., Ji C.J., Zhou X., Xia J.D., Song N.H. (2020). m6A RNA methylation regulators correlate with malignant progression and have potential predictive values in clear cell renal cell carcinoma. Exp. Cell Res..

[27] Zhang Y., Yao Y., Qi X., Li J., Liu M., Che X., Xu Y., Wu G. (2021). Identification of a New Prognostic Risk Signature of Clear Cell Renal Cell Carcinoma Based on N(6)-Methyladenosine RNA Methylation Regulators. J. Immunol. Res..

[28] Zheng Z., Mao S., Guo Y., Zhang W., Liu J., Li C., Yao X. (2020). N6-methyladenosine RNA methylation regulators participate in malignant progression and have prognostic value in clear cell renal cell carcinoma. Oncol. Rep..

[29] Zhong J., Liu Z., Cai C., Duan X., Deng T., Zeng G. (2021). M(6)A modification patterns and tumor immune landscape in clear cell renal carcinoma. J. Immunother. Cancer.

[30] Green N.H., Galvan D.L., Badal S.S., Chang B.H., LeBleu V.S., Long J., Jonasch E., Danesh F.R. (2019). MTHFD2 links RNA methylation to metabolic reprogramming in renal cell carcinoma. Oncogene.

[31] Zhang X., Wang F., Wang Z., Yang X., Yu H., Si S., Lu J., Zhou Z., Lu Q., Wang Z. (2020). ALKBH5 promotes the proliferation of renal cell carcinoma by regulating AURKB expression in an m(6)A-dependent manner. Ann. Transl. Med..

[32] Zhuang C., Zhuang C., Luo X., Huang X., Yao L., Li J., Li Y., Xiong T., Ye J., Zhang F. (2019). N6-methyladenosine demethylase FTO suppresses clear cell renal cell carcinoma through a novel FTO-PGC-1α signalling axis. J. Cell Mol. Med..

[33] Kang H., Zhang Z., Yu L., Li Y., Liang M., Zhou L. (2018). FTO reduces mitochondria and promotes hepatic fat accumulation through RNA demethylation. J. Cell Biochem..

[34] Marquardt A., Solimando A.G., Kerscher A., Bittrich M., Kalogirou C., Kübler H., Rosenwald A., Bargou R., Kollmannsberger P., Schilling B. (2021). Subgroup-Independent Mapping of Renal Cell Carcinoma-Machine Learning Reveals Prognostic Mitochondrial Gene Signature Beyond Histopathologic Boundaries. Front. Oncol..

[35] Moch H., Ulbright T., Humphrey P., Reuter V. (2016). WHO Classification of Tumours of the Urinary System and Male Genital Organs.

[36] The American Joint Commission on Cancer (2017). AJCC Cancer Staging Manual.

[37] Cerami E., Gao J., Dogrusoz U., Gross B.E., Sumer S.O., Aksoy B.A., Jacobsen A., Byrne C.J., Heuer M.L., Larsson E. (2012). The cBio cancer genomics portal: An open platform for exploring multidimensional cancer genomics data. Cancer Discov..

[38] Vieira J., Henrique R., Ribeiro F.R., Barros-Silva J.D., Peixoto A., Santos C., Pinheiro M., Costa V.L., Soares M.J., Oliveira J. (2010). Feasibility of differential diagnosis of kidney tumors by comparative genomic hybridization of fine needle aspiration biopsies. Genes Chromosomes Cancer.

[39] Jonasch E., Gao J., Rathmell W.K. (2014). Renal cell carcinoma. BMJ.

[40] Mitomi T., Kawahara T., Nomura S., Kuroda S., Takeshima T., Takamoto D., Otani M., Uemura H. (2020). Skin Metastasis of Renal Cell Carcinoma. Case Rep. Oncol..

[41] Shen H., Yang J., Huang Q., Jiang M.J., Tan Y.N., Fu J.F., Zhu L.Z., Fang X.F., Yuan Y. (2015). Different treatment strategies and molecular features between right-sided and left-sided colon cancers. World J. Gastroenterol. WJG.

[42] Wiener D., Schwartz S. (2021). The epitranscriptome beyond m(6)A. Nat. Rev. Genet..

[43] Meyer K.D., Jaffrey S.R. (2014). The dynamic epitranscriptome: N6-methyladenosine and gene expression control. Nat. Rev. Mol. Cell Biol..

[44] Peer E., Moshitch-Moshkovitz S., Rechavi G., Dominissini D. (2019). The Epitranscriptome in Translation Regulation. Cold Spring Harb Perspect. Biol..

[45] Sun T., Wu R., Ming L. (2019). The role of m6A RNA methylation in cancer. Biomed. Pharmacother..

[46] Roundtree I.A., Evans M.E., Pan T., He C. (2017). Dynamic RNA Modifications in Gene Expression Regulation. Cell.

[47] Shi H., Wei J., He C. (2019). Where, When, and How: Context-Dependent Functions of RNA Methylation Writers, Readers, and Erasers. Mol. Cell.

[48] Dango S., Mosammaparast N., Sowa M.E., Xiong L.J., Wu F., Park K., Rubin M., Gygi S., Harper J.W., Shi Y. (2011). DNA unwinding by ASCC3 helicase is coupled to ALKBH3-dependent DNA alkylation repair and cancer cell proliferation. Mol. Cell.

[49] Fedeles B.I., Singh V., Delaney J.C., Li D., Essigmann J.M. (2015). The AlkB Family of Fe(II)/α-Ketoglutarate-dependent Dioxygenases: Repairing Nucleic Acid Alkylation Damage and Beyond. J. Biol. Chem..

[50] Tao L., Mu X., Chen H., Jin D., Zhang R., Zhao Y., Fan J., Cao M., Zhou Z. (2021). FTO modifies the m6A level of MALAT and promotes bladder cancer progression. Clin. Transl. Med..

[51] Thomas J.M., Batista P.J., Meier J.L. (2019). Metabolic Regulation of the Epitranscriptome. ACS Chem. Biol..

[52] Deng X., Su R., Stanford S., Chen J. (2018). Critical Enzymatic Functions of FTO in Obesity and Cancer. Front. Endocrinol..

[53] Yu H., Zhang Z. (2021). ALKBH5-mediated m6A demethylation of lncRNA RMRP plays an oncogenic role in lung adenocarcinoma. Mamm. Genome.

[54] Zhang J., Guo S., Piao H.Y., Wang Y., Wu Y., Meng X.Y., Yang D., Zheng Z.C., Zhao Y. (2019). ALKBH5 promotes invasion and metastasis of gastric cancer by decreasing methylation of the lncRNA NEAT1. J. Physiol. Biochem..

[55] Han Z., Wang X., Xu Z., Cao Y., Gong R., Yu Y., Yu Y., Guo X., Liu S., Yu M. (2021). ALKBH5 regulates cardiomyocyte proliferation and heart regeneration by demethylating the mRNA of YTHDF1. Theranostics.

[56] Huff S., Tiwari S.K., Gonzalez G.M., Wang Y., Rana T.M. (2021). M(6)A-RNA Demethylase FTO Inhibitors Impair Self-Renewal in Glioblastoma Stem Cells. ACS Chem. Biol..

[57] Malacrida A., Rivara M., Di Domizio A., Cislaghi G., Miloso M., Zuliani V., Nicolini G. (2020). 3D proteome-wide scale screening and activity evaluation of a new ALKBH5 inhibitor in U87 glioblastoma cell line. Bioorg. Med. Chem..

[58] Li J., Han Y., Zhang H., Qian Z., Jia W., Gao Y., Zheng H., Li B. (2019). The m6A demethylase FTO promotes the growth of lung cancer cells by regulating the m6A level of USP7 mRNA. Biochem. Biophys. Res. Commun..

[59] Niu Y., Lin Z., Wan A., Chen H., Liang H., Sun L., Wang Y., Li X., Xiong X.F., Wei B. (2019). RNA N6-methyladenosine demethylase FTO promotes breast tumor progression through inhibiting BNIP3. Mol. Cancer.

[60] Zou D., Dong L., Li C., Yin Z., Rao S., Zhou Q. (2019). The m(6)A eraser FTO facilitates proliferation and migration of human cervical cancer cells. Cancer Cell Int..

[61] Shen C., Sheng Y., Zhu A.C., Robinson S., Jiang X., Dong L., Chen H., Su R., Yin Z., Li W. (2020). RNA Demethylase ALKBH5 Selectively Promotes Tumorigenesis and Cancer Stem Cell Self-Renewal in Acute Myeloid Leukemia. Cell Stem Cell.

[62] Thomsen M.B.H., Nordentoft I., Lamy P., Vang S., Reinert L., Mapendano C.K., Høyer S., Ørntoft T.F., Jensen J.B., Dyrskjøt L. (2017). Comprehensive multiregional analysis of molecular heterogeneity in bladder cancer. Sci. Rep..

[63] Aran D., Camarda R., Odegaard J., Paik H., Oskotsky B., Krings G., Goga A., Sirota M., Butte A.J. (2017). Comprehensive analysis of normal adjacent to tumor transcriptomes. Nat. Commun..

[64] Xiao Y., Thakkar K.N., Zhao H., Broughton J., Li Y., Seoane J.A., Diep A.N., Metzner T.J., Von Eyben R., Dill D.L. (2020). The m(6)A RNA demethylase FTO is a HIF-independent synthetic lethal partner with the VHL tumor suppressor. Proc. Natl. Acad. Sci. USA.

