# Supplementary material for: Deregulation of N6-Methyladenosine RNA Modification and Its Erasers FTO/ALKBH5 among the Main Renal Cell Tumor Subtypes
Source: J Pers Med. 2021 Sep 30;11(10):996. doi: 10.3390/jpm11100996 (PMC8538585; doi:10.3390/jpm11100996)
Supplement: Supplementary file 1 [file jpm-11-00996-s001.zip › jpm-1336651-supplementary.pdf]

Supplementary Tables

Table S1. Clinicopathological characteristics of IPO Porto cohort

Clinicopathological features	Patients (n= 160)
**Median age, years**	61 (29-86)
**Gender, n (%)**	
**Male**	97 (60.6)
**Female**	63 (39.4)
**Histological type**	
Clear cell RCC	40/160
Papillary RCC	40/160
Chromophobe RCC	40/160
Oncocytoma	40/160
**Pathological stage**	
I	67/120
II	24/120
III	25/120
IV	4/120

Table S2. Univariable analysis in TCGA ccRCC patients

	Overall Survival			Progression Free Survival		
**Variable**	**HR**	**95% IC**	**p-value**	**HR**	**95% IC**	**p-value**
**FTO expression**						
≤ P25	1.00	-	-	1.00	-	-
> P25	**0.61**	**0.39-0.95**	**0.027**	**0.60**	**0.39-0.93**	**0.024**
**ALKBH5 expression**						
≤ P75	1.00	-	-	1.00	-	-
> P75	1.47	0.95-2.28	0.080	1.53	0.98-2.37	0.060
**Sex**						
Female	1.00	-	-	1.00	-	-
Male	0.80	0.53-1.21	0.285	1.23	0.80-1.90	0.340
**Age at diagnosis**	**1.04**	**1.02-1.06**	**< 0.001**	1.01	0.99-1.03	0.185
**Stage**						
T1	1.00	-	-	1.00	-	-
T2	1.19	0.59-2.39	0.6288	3.29	1.71-6.34	**< 0.001**
T3/T4	**3.23**	**2.05-5.10**	**< 0.001**	6.09	3.62-10.26	**< 0.001**

Table S3. Univariable analysis in TCGA pRCC patients

	Overall Survival			Progression Free Survival		
**Variable**	**HR**	**95% IC**	**p-value**	**HR**	**95% IC**	**p-value**
**FTO expression**						
≤ P25	1.00	-		1.00	-	
> P25	**0.49**	**0.25-0.95**	**0.035**	**0.48**	**0.27-0.84**	**0.010**
**ALKBH5 expression**						
≤ P75	1.00	-		1.00	-	
> P75	1.04	0.51-2.11	0.913	1.19	0.65-2.18	0.582
**Sex**						
Female	1.00	-		1.00	-	
Male	0.79	0.37-1.68	0.537	0.60	0.33-1.11	0.105
**Age at diagnosis**	1.01	0.98-1.04	0.550	0.99	0.97-1.01	0.429
**Stage**						
T1	1.00	-		1.00	-	
T2	**3.40**	**1.23-9.37**	**0.018**	**3.63**	**1.61-8.14**	**0.002**
T3/T4	**8.17**	**3.79-17.59**	**< 0.001**	**6.84**	**3.65-12.81**	**< 0.001**

Table S4. Univariable analysis in TCGA chRCC patients

	Overall Survival			Progression Free Survival		
**Variable**	**HR**	**95% IC**	**p-value**	**HR**	**95% IC**	**p-value**
**FTO expression**						
≤ P25	1.00	-		1.00	-	
> P25	0.39	0.10-1.44	0.157	0.35	0.11-1.16	0.085
**ALKBH5 expression**						
≤ P75	1.00	-		1.00	-	
> P75	2.79	0.75-10.4	0.126	2.86	0.87-9.38	0.083
**Sex**						
Female	1.00	-		1.00	-	
Male	1.56	0.39-6.23	0.532	0.95	0.29-3.11	0.930
**Age at diagnosis**	**1.06**	**1.00-1.12**	**0.049**	1.04	0.99-1.09	0.111
**Stage**						
T1	1.00	-		1.00	-	
T2	-	-	-	1.78	0.16-19.59	0.639
T3/T4	-	-		**11.93**	**1.49-95.74**	**0.020***

Supplementary Figures

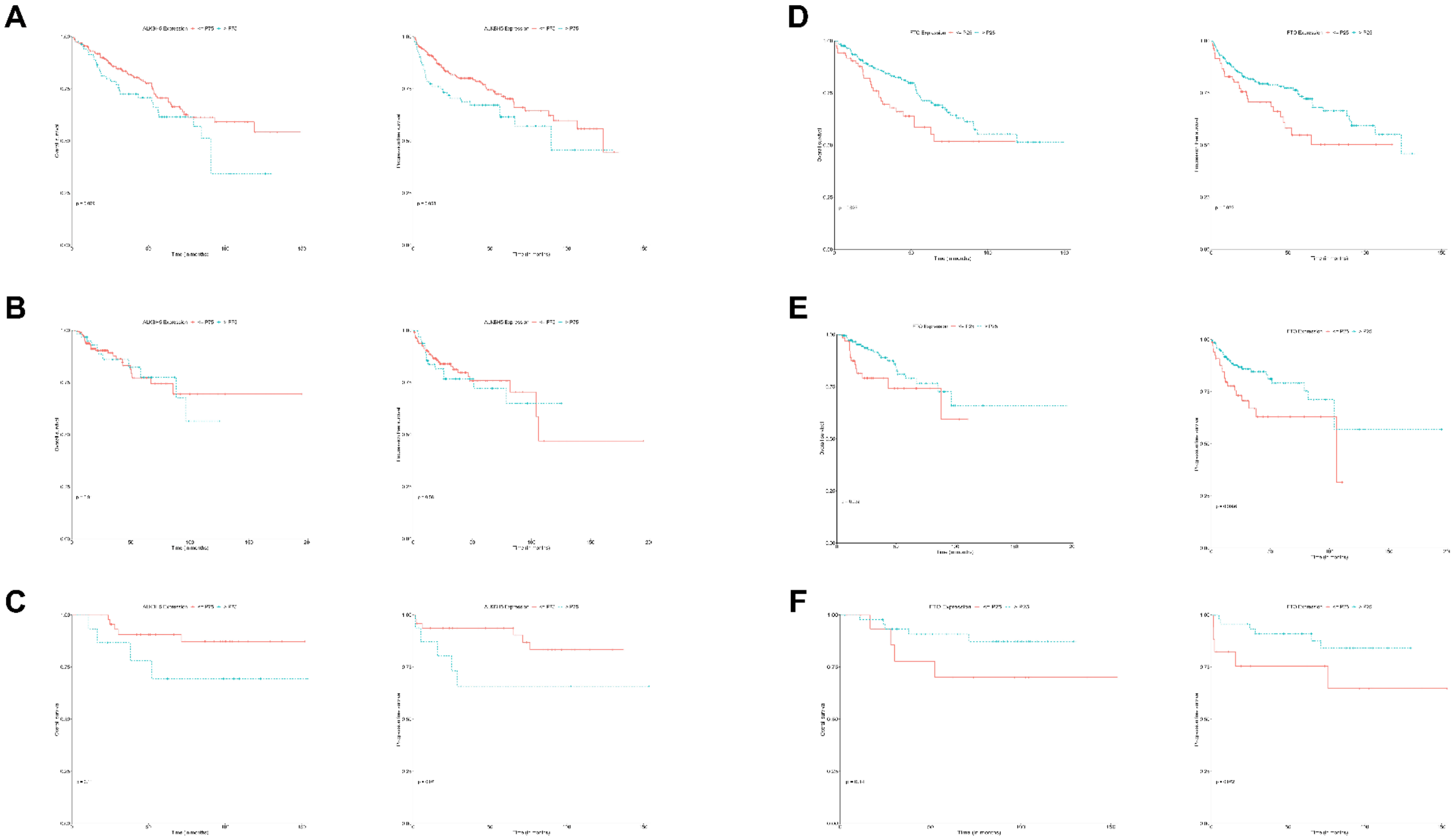

Figure S1. Survival analysis in TCGA's patients for **ALKBH5** (A) Overall-survival (left) and Progression free-survival (right) in ccRCC (B) Overall-survival (left) and Progression free-survival (right) in pRCC (C) Overall-survival (left) and Progression free-survival (right) in chRCC **and FTO** (D) Overall-survival (left) and Progression free-survival (right) in ccRCC (E) Overall-survival (left) and Progression free-survival (right) in pRCC (F) Overall-survival (left) and Progression free-survival (right) in chRCC.

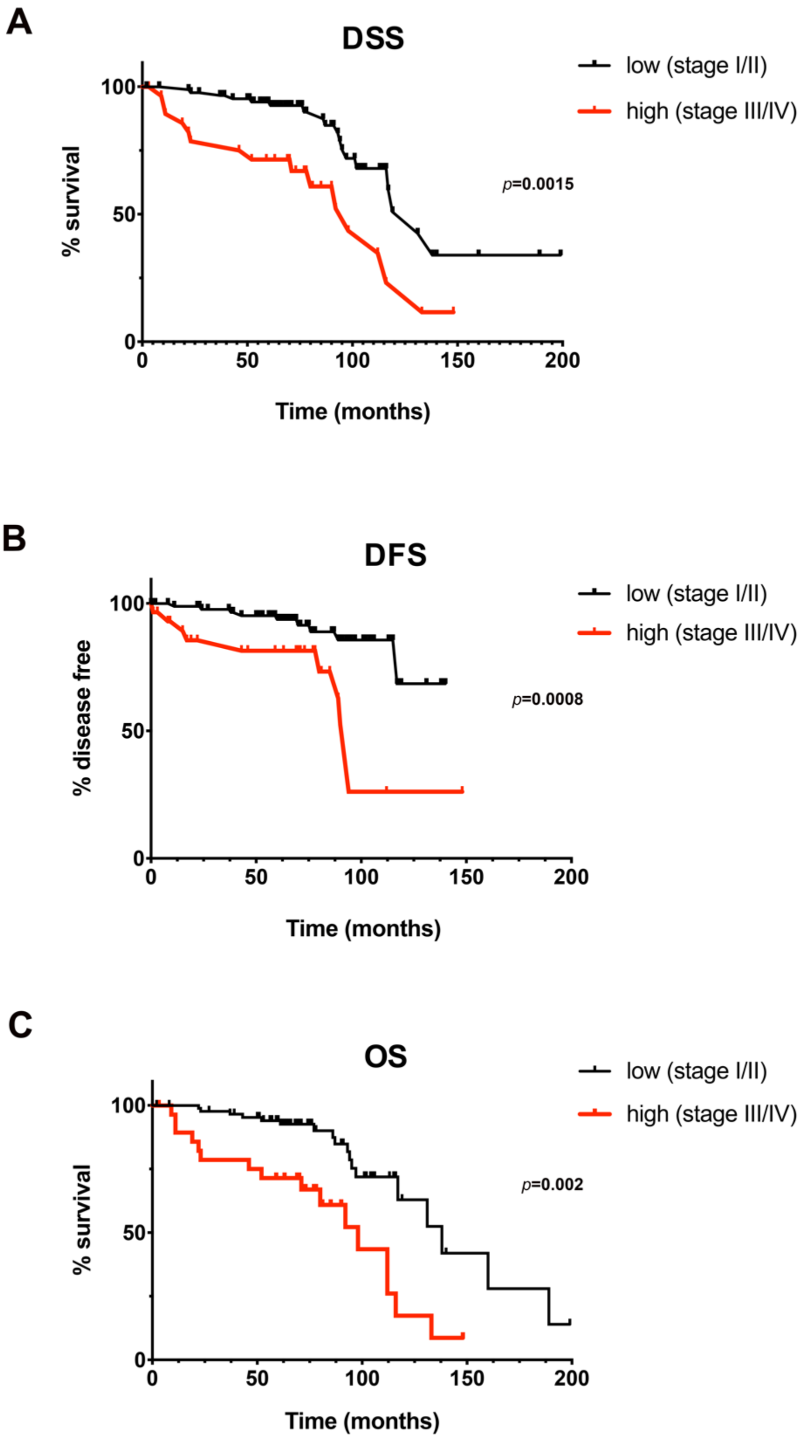

Figure S2. Kaplan-Meier estimated (A) Disease-specific survival (B) Disease-free survival (C) Overall survival for stage of IPO Porto's cohort.

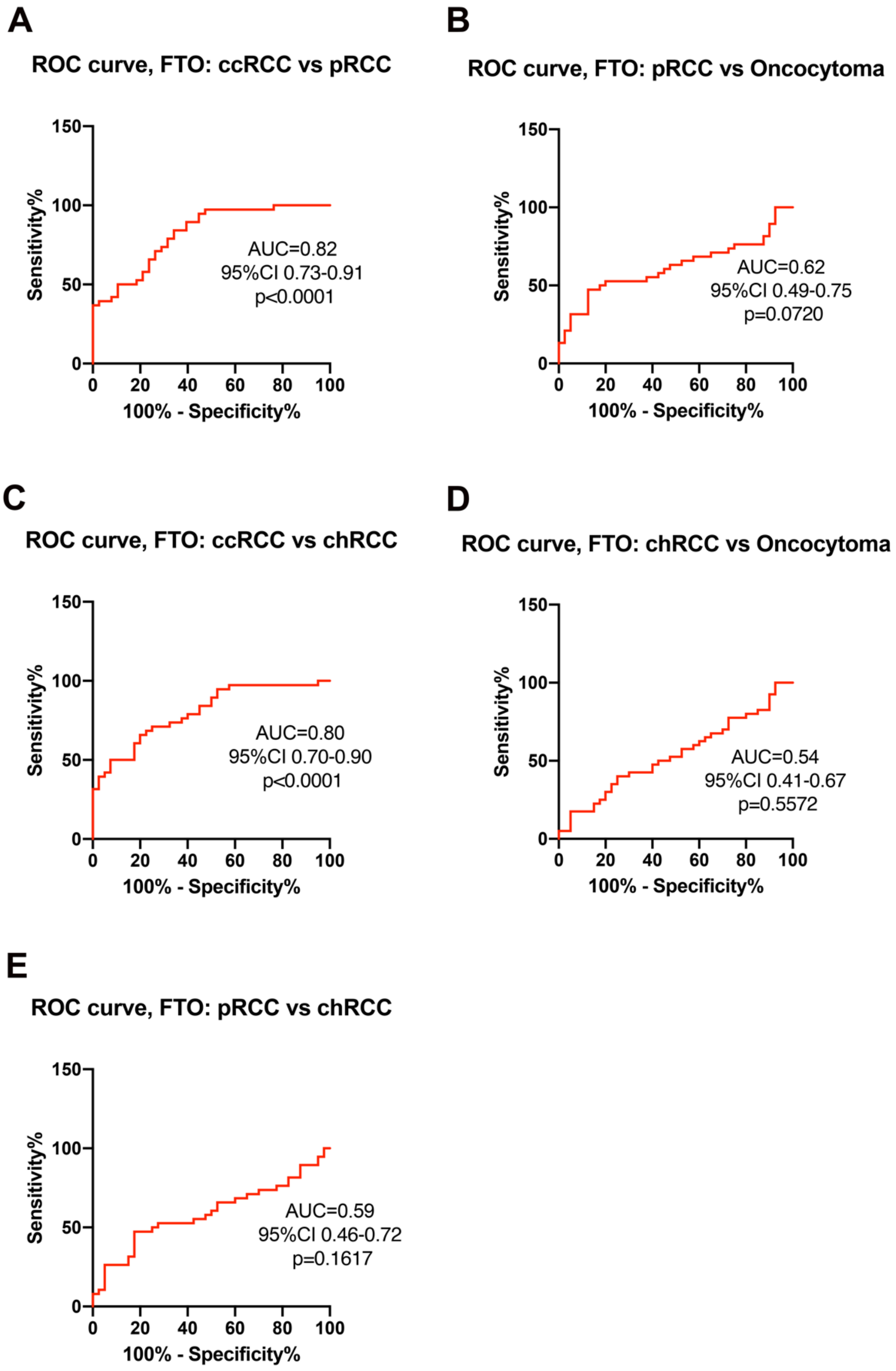

Figure S3. Transcript levels of FTO: ROC curve for discrimination among different subtypes based on FTO mRNA expression levels. Abbreviations: ccRCC- clear cell renal cell carcinoma; pRCC- papillary renal cell carcinoma; chRCC- chromophobe renal cell carcinoma; AUC – Area under the curve; CI – Confidence interval.

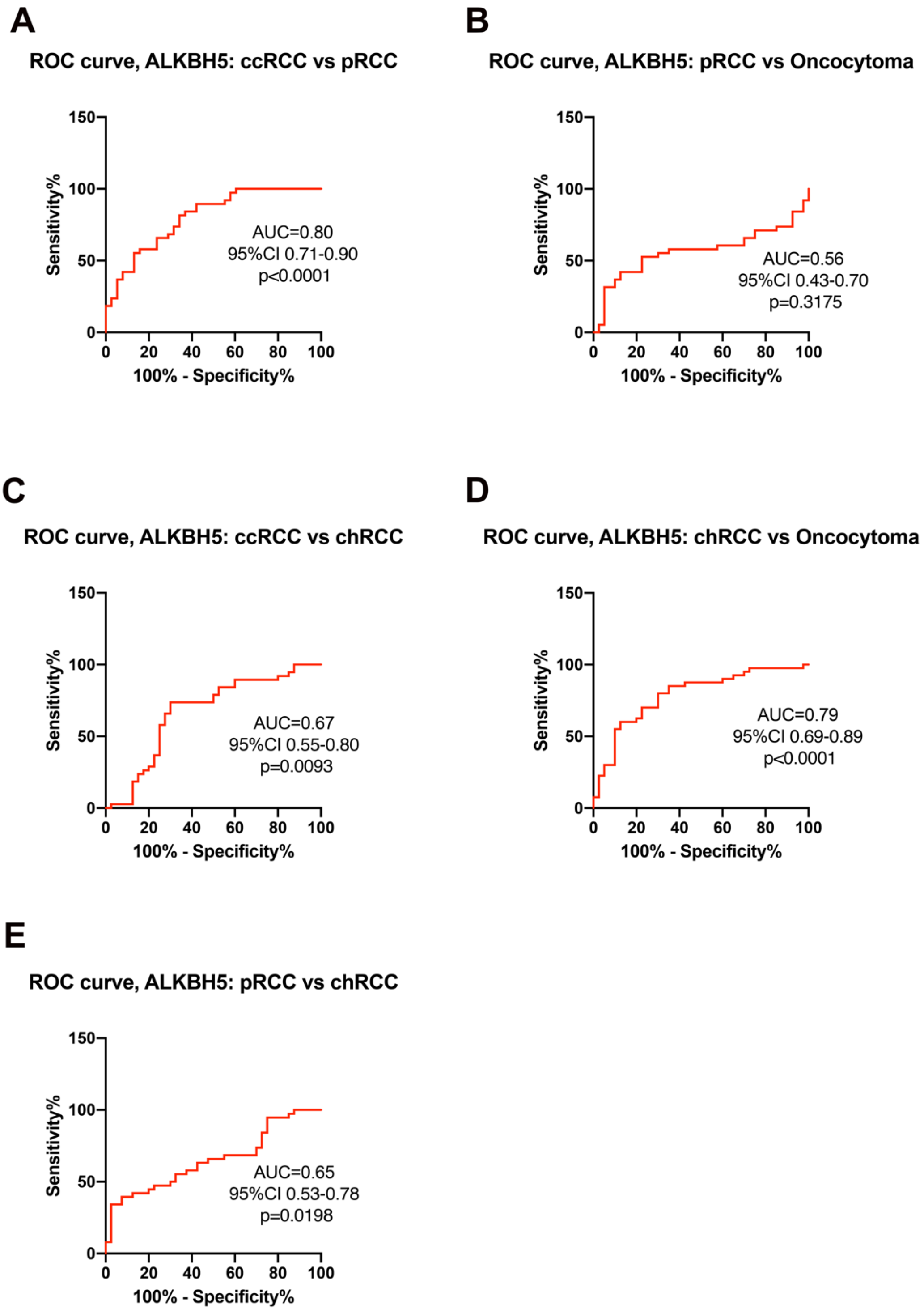

Figure S4. Transcript levels of ALKBH5**:** ROC curve for discrimination among different subtypes based on ALKBH5 mRNA expression levels. Abbreviations: ccRCC- clear cell renal cell carcinoma; pRCC- papillary renal cell carcinoma; chRCC- chromophobe renal cell carcinoma; AUC – Area under the curve; CI – Confidence interval**.**

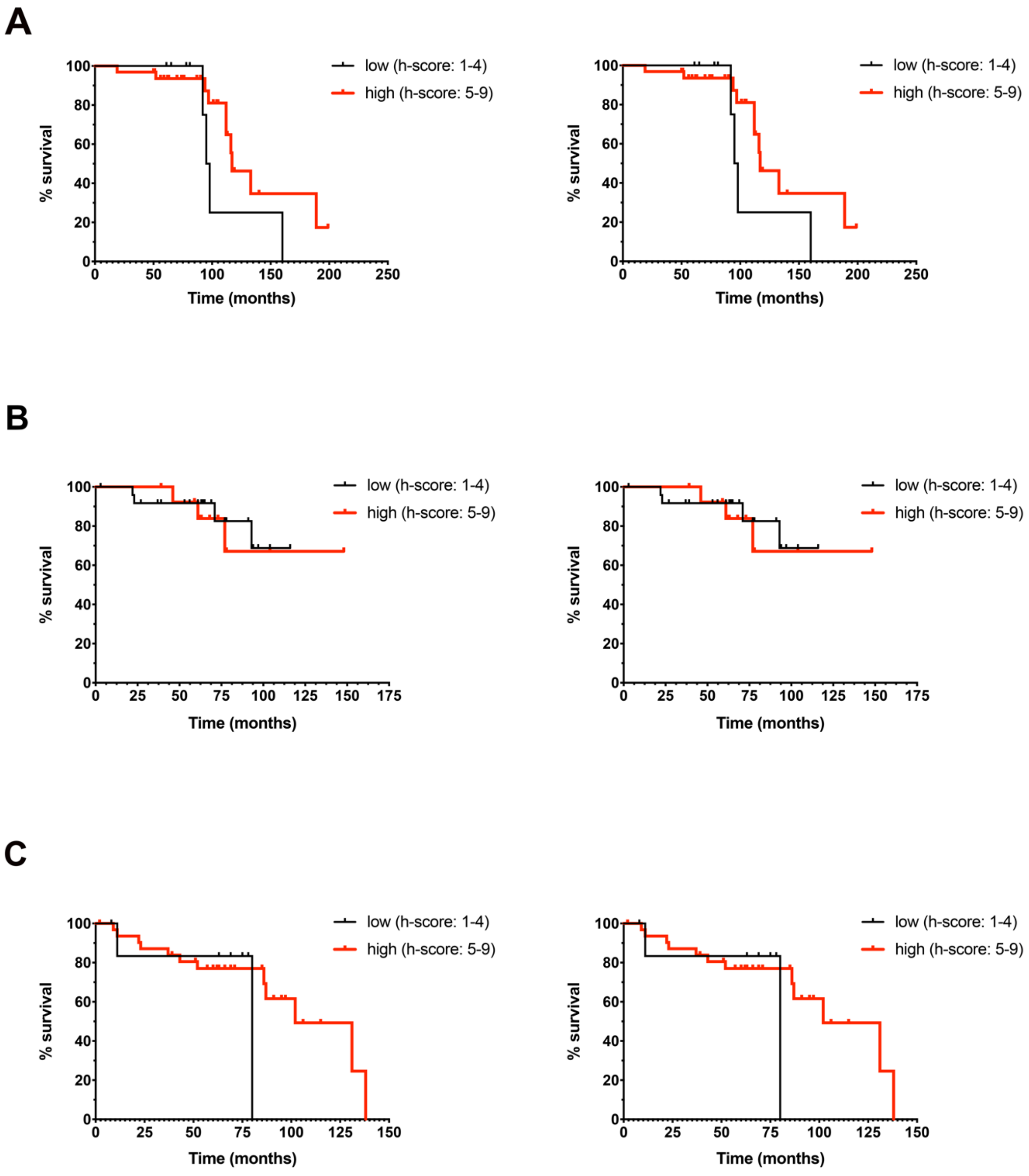

Figure S5. Kaplan-Meier estimated (A) Overall-survival in ccRCC (B) Overall-survival in chRCC (C) Overall survival in pRCC for FTO (right) and ALKBH5 (left) immunoexpression in IPO Porto cohort.
